# Book Reviews

**DOI:** 10.1155/S146392467900064X

**Published:** 1979


### Book Reviews

### Topics in Automatic Chemical Analysis Edited by J.K. Foreman and P.B. Stockwell

*Ellis Horwood, Chichester, 1979. pp 311, £21.50 ISBN 0-85312-079*

This book is the first of a series planned by the editors to follow their previous book 'Automatic Chemical Analysis'. It is intended to inform the readers of the current state of the art, progress and developments made in this field and is presented in the form of nine chapters written by acknowledged experts in the development and application of automation in analysis. As such, there are obviously a variety of styles and the chapters range from those of a review nature to direct and very full descriptions of particular analysers. The book is well written, clearly presented and in all respects is easy to read. To the experienced worker in the field it brings together some recent work and to the newcomer provides some useful examples of what can be done.

The first chapter, written by the editors, deals with the 'Philosophy and Practice of Automatic Analysis'. This provides a useful introduction to the other contributions and recalls the definitions relating to automation, the needs, the advantages, the limitations and many of the ways and means. Useful examples are presented and the authors rightly discuss such important aspects as reliability, maintenance, readjustment of staff expertise, education and training, the requirements for defining the analytical specification, and above all, the continuing importance of the analyst.

The second chapter by D.G. Porter and P.B. Stockwell covers the 'In-house Design and Construction of Automatic Analysers for Laboratory Use'. This chapter continues the philosophical discussions started in chapter 1, and additionally provides discussion about instrument specifications related to analytical objectives, 'build it yourself' assemblies and modification of commercial equipment. The importance of a proper evaluation at the prototype stage is emphasised and the essential features covering the various categories of hardware modules, ranging from pumps to the data processing units, are discussed. Again specific examples are provided to demonstrate the points made.

Chapter 3, written by R.W. Arndt and R.D. Werder, covers 'Automated Individual Analysis in Wet Chemistry Laboratories'. Again the important philosophical points are made particularly those relating to the need to consider the whole system and for the need for the operator to retain responsibility. The author deals with procedure and compares the needs and operations carried out in the clinical laboratory with those in the industrial laboratory. The elements for automatic analysis including basic operation, sample transport, central control, entry and weighing stations, output station and logistics are discussed, together with control requirements for single and multiple stage operations. The system concept is finally demonstrated by reference to a titrator application.

Chapter 4 deals with the more specialised area of 'Automated Reaction Rate Methods of Analysis'. The authors, H.P. Malmstadt, D.L. Krottinger and M.S. McCracken, point out that such methods become a practical proposition because of automation and the constant reaction conditions this provides. Rapid results can be obtained at intermediate reaction rates and advantage taken of the selectivity of enzyme and other reactions provided interfering reactions are slow in comparison. Aspects of the analytical protocols and instrument modules are discussed in some detail. These include sample and reagent preparation, reaction temperature, electrochemical and spectroscopic measurement, rate information, calculation and control. Specific automated systems are then described.

The 'Application of the Technicon Auto-Analyser II to the Water soluble Vitamins in Foodstuffs' is described by R.B. Roy in chapter 5. Methods for thiamin, riboflavine niacin, niacinamide and ascorbic acid are described and used to demonstrate the author's point that a good understanding of the chemistry is required to get the best from an automated procedure.

In a very useful chapter 6 by H.L. Pardue, the 'Applications of Imaging Detectors for Analytical Spectroscopy' are described. The various type of imaging detector together with their functions and methods of application in the fields of absorption spectroscopy, fluorescence and atomic spectroscopy, derivative spectroscopy, Raman spectroscopy and mass spectrometry are discussed. The advantages and disadvantages are usefully illustrated by examples and the summary remarks clearly give the present state of the art in this area.

J. Bierens de Haan presents a comprehensive 'Critique of Automated Methods in Clinical Analysis' in chapter 7 and gives the history of past and present instrumentation providing principles, advantages and limitations of large and fully automated systems, the smaller and more flexible installations and the specialised push button instruments. Use is made of the IUPAC recommendations in a systematic survey outlining where gaps exist in the existing technology. This author also underlines the need for physchological acceptance, clear definition of requirements, self monitoring self correcting instruments but with the overriding need for communication facilities to the controlling operator, together with the availability of basic data. Poor analytical methods or sub optimum conditions should not be used for the convenience of engineering procedures. An appendix detailing some of the terminology used by the International Federation of Clinical Chemistry is provided.

In chapter 8 D.R. Deans and R.M. Peterson present an invaluable contribution on 'The Application of Automation to Quantitative Gas Chromatography in Petrochemical Analysis'. This chapter presents a wealth of good advice to anyone contemplating the use of automated gas chromatography. It covers the control and flow switching systems and gives examples of optimisation in design. The author underlines the need for modular design and makes the point that a mechanical system can never match the versatility of the human operator. Many practical aspects of the hardware and operation are discussed, including valves, autosamplers, calibration procedures, sample identification methods. The chapter contains a useful section on data processing covering such important features as nature and format of the analytical report and validity checks, and concludes with some thoughts on user constructed systems, control requirements and the interaction with larger computers.

The final chapter by G.K.E.Copeland and P.B. Stockwell, describes their experience in 'Automating Large-Scale Routine Analysis of Cigarette Smoke'. This chapter gives a detailed description of the developments in the analytical systems for the analysis of cigarette smoke for the Department of Health and Social Security requirements. It provides an excellent example of the development of a project through the stages of defining the requirements, setting specifications to the methods and instrumentation and working through the manual and semi-automated systems with off-line data handling through to complete automation and on-line computing. A multi-discipline team of analysts and automation experts provides facilities for control of sampling and sub-sampling, and the subsequent determination of carbon monoxide, water, tar and nicotine in smoke produced from an automated smoking machine.

*D.C.M. Squirrell*

